# Mismatched anti-predator behavioral responses in predator-naïve larval anurans

**DOI:** 10.7717/peerj.1472

**Published:** 2015-12-07

**Authors:** Molly Albecker, Heather D. Vance-Chalcraft

**Affiliations:** Department of Biology and East Carolina Biodiversity Initiative, East Carolina University, Greenville, NC, United States

**Keywords:** Antipredator behavior, Innate risk detection, Predation risk, Predator efficiency, Anuran amphibian

## Abstract

Organisms are adept at altering behaviors to balance the tradeoff between foraging and predation risk in spatially and temporally shifting predator environments. In order to optimize this tradeoff, prey need to be able to display an appropriate response based on degree of predation risk. To be most beneficial in the earliest life stages in which many prey are vulnerable to predation, innate anti-predator responses should scale to match the risk imposed by predators until learned anti-predator responses can occur. We conducted an experiment that examined whether tadpoles with no previous exposure to predators (i.e., predator-naive) exhibit innate antipredator behavioral responses (e.g., via refuge use and spatial avoidance) that match the actual risk posed by each predator. Using 7 treatments (6 free-roaming, lethal predators plus no-predator control), we determined the predation rates of each predator on *Lithobates sphenocephalus* tadpoles. We recorded behavioral observations on an additional 7 nonlethal treatments (6 caged predators plus no-predator control). Tadpoles exhibited innate responses to fish predators, but not non-fish predators, even though two non-fish predators (newt and crayfish) consumed the most tadpoles. Due to a mismatch between innate response and predator consumption, tadpoles may be vulnerable to greater rates of predation at the earliest life stages before learning can occur. Thus, naïve tadpoles in nature may be at a high risk to predation in the presence of a novel predator until learned anti-predator responses provide additional defenses to the surviving tadpoles.

## Introduction

Predation is an important force structuring communities, especially in groups of concern such as amphibians (Sih et al., 1985; Lima & Dill, 1990; Abrams, 1995; Lima, 1998; Werner & Peacor, 2003; Bolker et al., 2003; Preisser, Bolnick & Benard, 2005). Within anurans, the egg and larval stages are very vulnerable to predation (Alford, 1999; Laurila, Karttunen & Merilä, 2002) so there is considerable interest in understanding predation at these early life stages. Even though the majority of North American anuran species do not display extended parental care by caring for egg clutches/tadpoles following egg deposition, mothers may influence the likelihood that their offspring will encounter predators during early life stages through oviposition site selection (Resetarits Jr & Wilbur, 1989; Blaustein et al., 2004; Rieger, Binckley & Resetarits, 2004). However, in some systems, predator composition is highly variable and can change suddenly before the young are past their most vulnerable stages. Therefore, it is adaptive for offspring to have defenses to a variety of potential predators from the moment of hatching if the survival benefits (e.g., increased foraging opportunities) afforded by anti-predator defenses are not offset by costs in other areas of fitness, such as reduced competitive ability or limited foraging opportunities (Bolker et al., 2003; Werner & Peacor, 2003; McPeek, 2004). Indeed, for prey to optimize the tradeoff between costs and benefits, they need the innate ability to recognize specific predators and the risk they impose, and the ability to respond appropriately based on this risk (Helfman, 1989; Chivers et al., 2001; Walzer & Schausberger, 2013).

Responses to the threat of predation can be either innate, where the response results from inherent, intrinsic genetic or physiological underpinnings, or learned, which typically arise following exposure to predator-derived kairomones, consumption-generated alarm cues, or other stimuli (Ferrari, Wisenden & Chivers, 2010). In aquatic habitats, the transmission and detection of chemical cues have been implicated as the primary signal by which tadpoles and other aquatic taxa recognize the riskiness of their surroundings (Semlitsch & Gavasso, 1992; Werner & Anholt, 1996; Eklöv, 2000; Barros et al., 2002; Schoeppner & Relyea, 2005; Fraker, 2009). Innate predator responses are evolved reactions whereby prey can diagnose the level of threat present in the environment without any prior direct experience with the predator (Blaustein et al., 1997; Epp & Gabor, 2008; DeSantis, Davis & Gabor, 2013; Stratmann & Taborsky, 2014). These responses are typically observed during young age classes and when new predators are introduced (Murray, Roth & Wirsing, 2004; Gall & Mathis, 2010). In contrast, with learned predator responses, prey generate or adjust antipredator behaviors following encounters and experiences with predators (Woody & Mathis, 1998; Mirza et al., 2006; Ferrari et al., 2007; Ferrari, Messier & Chivers, 2007).

Studies suggest that the subsequent response of prey to a predator should reflect the amount of predator-induced risk (e.g., threat sensitivity hypothesis *sensu* Helfman, 1989) (Helfman, 1989; Lima & Dill, 1990; Abrams & Matsuda, 1996; Peacor & Werner, 2004; McCoy, 2007). Anuran tadpoles and their predators have been models for studying predator-induced responses, and many species have been documented to display hierarchical responses according to the perceived dangerousness of the predator (Relyea & Werner, 1999; Relyea, 2000; Relyea, 2003; Teplitsky, Plenet & Joly, 2004; Ferrari, Sih & Chivers, 2009). Support for the threat sensitivity hypothesis also has been found for larval salamanders (Mathis, Murray & Hickman, 2003), arthropods (Walzer & Schausberger, 2013), and fish (Brown & Smith, 1998), as well as other taxa. Notably, this hypothesis does not discriminate between innate and learned predator responses; rather it generally predicts that all responses to predators should be scaled according to perceived threat. However, prey that have evolved innate anti-predator responses may be able to respond according to threat during the most vulnerable early life stages, while prey that rely primarily on learned responses may lack this ability until after they have had encounters with predator cues.

We conducted an experiment that examined whether tadpoles with no previous exposure to predators (i.e., predator-naive) exhibit innate antipredator behavioral responses (e.g., via refuge use and spatial avoidance) that match the actual risk posed by each predator based upon consumption. We focus on refuge use and spatial avoidance responses as these behaviors are instantaneous, labile, and often compose the first line of defense compared to the more gradual responses such as morphological changes (Chivers & Smith, 1998; Relyea, 2001). Because tadpoles are often found in habitats with variable and changing predator communities, we predicted that tadpoles would rely more on learned responses and thus would not display innate responses that match to relative threat imposed by predators. We expect that the magnitude of the initial behavioral responses of naïve prey to different predators will not match the risk imposed by the predators. If our hypothesis is supported, newly hatched or predator-naïve tadpole populations may be vulnerable to shifting or novel predator communities in nature. Indeed, numerous examples exist where amphibians were unable to recognize an introduced predator as a threat, leading to entire prey populations being decimated (Gamradt & Kats, 1996; Gillespie, 2001; Kats & Ferrer, 2003).

## Materials and Methods

### Study system

We used Southern leopard frog tadpoles (*Lithobates sphenocephalus*, formerly *Rana sphenocephala*) to test innate predator responses. This species is abundant in temporary pond systems across the Southeastern United States characterized by variable and shifting predator communities. Six natural predator species, known to consume and co-occur with *L. sphenocephalus*, were chosen to represent a range of possible predator encounters in natural wetland systems. We chose dragonfly larvae (*Pachydiplax longipennis*), the white crayfish (*Procambarus acutus*), pirate perch (*Aphredoderus sayanus*), bluegill sunfish (*Lepomis macrochirus*), the broken-striped newt (*Notophthalmus viridescens viridescens*), and a fishing spider (*Dolomedes triton*). These predators encapsulate a variety of predator characteristics found naturally in southeastern aquatic habitats and represent ambush and pursuit predators, tactile and visual predators, as well as benthic and pelagic predators.

We collected freshly laid clutches of *L. sphenocephalus* eggs from the Croatan National Forest and from small wetlands surrounding Greenville, North Carolina on 18 May 2010 and again on 12 July 2010. All experimental tadpoles were between Gosner stages 26 and 30 and within the consumable size range for the gape limited predators (Gosner, 1960). The predators were collected from the Croatan National Forest, North Carolina, and Bray Hollow in Grifton, North Carolina. The crayfish, spiders, and dragonfly larvae were housed in separate 1 L containers and fed 2 tadpoles daily. The pirate perch, sunfish, and newts were held in separate aquaria (57 L). The fish and newt predators were fed *L. sphenocephalus* tadpoles *ad libitum*. Each predator was released back to its original habitat within seven days of being used in a trial. All procedures in this study were designed to minimize animal distress and mortality. This study was approved by the University of East Carolina’s Institute for Animal Care and Use Committee (Animal Use Protocol #D246). Animals were collected under North Carolina Wildlife Resources Commission Scientific Collection Permit (-SC00232).

### Experimental methods

The tadpole-survival component of the experiment consisted of 7 treatments (6 free-roaming, lethal predators plus no-predator control) and the behavioral component of the experiment consisted of 7 nonlethal treatments (6 caged predators plus no-predator control). The experiments were run concurrently. The no-predator controls and the lethal predator treatments each contained an empty cage identical to those used in the non-lethal behavioral treatments. Each treatment was replicated once in each of eight temporal blocks that took place over an eight-week period. Similarly sized tadpoles were used within a block. Each experimental tank received 20 tadpoles and was randomly assigned to a predator treatment. Experiments were conducted in glass aquariums (57 L; length × width × height: 61.0 × 30.5 × 30.5 cm) in a laboratory with barriers between aquaria to prevent visual interactions between organisms in different tanks. All tanks received natural light for 14.5 h (±3 min) per day. Aquaria were marked 7.6 cm above the bottom and marked again 7.6 cm above that line to form three equal-sized, distinct regions in the water column. Each tank was filled with 43.5 L of dechlorinated tap water (TopFin^®^ Tap Water Dechlorinator). Ten grams of fresh mixed deciduous and pine leaf litter was added to each tank to provide refuge and food resources for the tadpoles. The mixed litter was stirred in the aquarium, allowed to settle, and typically covered approximately 70% of the bottom of the aquaria with a max litter depth of 2.5 cm. Litter from evergreen and deciduous canopies is a common substrate in ponds and lakes along the coastal plain of North Carolina, and was chosen to provide additional realism for predators and prey. Predator cages were made from clear plastic containers (15.2 cm × 15.2 cm × 25.4 cm) covered on two sides with mesh screen to allow water and chemical cues to freely circulate.

To begin each temporal block, groups of twenty *L. sphenocephalus* tadpoles were haphazardly selected, placed into each tank, and allowed to acclimatize for an hour. Tanks were then randomly assigned to one of the predator treatments. Predators were weighed and measured after being starved for 36 h prior to the start of the trial (Table 1). Caged predators were all fed five similarly sized tadpoles in their cages at the beginning of each block to ensure that the amount of cue (consumed tadpoles) present in all treatments was similar (Laurila, Kujasalo & Ranta, 1997; McCoy et al., 2012). To ensure that cages did not impede tadpole observations, all cages were placed at the rear of the tanks.

**Table 1:** Predator information. The mean length (in mm), standard deviation of the length (in mm), mean mass (in g), and standard deviation of the mass (in g) of all predators used during the experiment. The average length of the fish predators (bluegill sunfish and pirate perch) is the standard length from the tip of the snout to the end of the caudal peduncle. The length of the cephalothorax and abdomen comprise the average length of the fishing spider. The dragonfly larvae were similarly measured from its head to the end of the abdomen. The length of the white crayfish was measured from the cephalothorax and abdomen (excluding the telson). The length of the red striped newt is the length from the snout to the vent. The mean mass is the average weight of each predator.

	Mean length (mm)	Std. dev. (mm)	Mean mass (g)	Std. dev. (g)
Bluegill sunfish	53.03	6.06	4.38	1.8
Pirate perch	60.29	7.96	5.18	1.95
Fishing spider	11.12	1.20	0.73	0.31
White crayfish	59.33	5.32	8.24	1.69
Dragonfly larvae	20.17	2.88	0.42	0.13
Red striped newt	35.96	5.38	2.12	0.32

In the survivorship treatments (i.e., free-roaming predators), the predators were removed from the tanks twenty hours after being placed in the aquaria, and number of surviving prey was recorded. No predators were used more than once in the experiment. In the nonlethal treatments, we observed each tank for tadpole position and visibility twice during each temporal block–approximately two hours after the predators were added, and again prior to the termination of the experiment (at 20 h). We chose to end trials at 20-hours in an effort to balance the opposing needs of acquiring a snapshot of naïve tadpole behaviors prior to learning, while also allowing sufficient time for the less lethal or nocturnal predators to consume prey. Observations occurred in the late afternoon and again at least 16 h later prior to the trial termination. During each of the two observation periods (early and late), the observer sat for a 5-minute acclimation period and maintained a distance of approximately 30 cm from the aquarium throughout the observation period. We recorded the number of tadpoles visible on the bottom third of the tank, in the middle third, and in the top third of the tank at thirty-second intervals for 10 min. We use tadpole visibility as a proxy for refuge use as visible tadpoles were assumed to represent those tadpoles not hiding within the leaf litter. To quantify the spatial positions of tadpoles, we counted the number using the top two thirds of the tank and quantified the number on the bottom by subtracting the number on the top from 20 (the total number of prey per tank). Thus, we had two behavioral response variables, refuge use and spatial position.

### Data analysis

Tadpole survivorship in the lethal predator treatments was tested using a generalized linear mixed effects model (GLMM) with a binomial family error distribution using package blme (Dorie, 2014) in the R statistical programming environment (R Core Development Team, 2014). The Bayesian linear mixed-effects model corrects for bias resulting from complete separation in binomial responses that we observed in the no-predator control treatments (Gelman et al., 2008; Kosmidis, 2013; Dorie, 2014). Predator identity was treated as a fixed effect, while temporal block was treated as a random effect. We used false discovery rate (Benjamini & Hochberg, 1995) corrected post hoc pairwise comparisons to test for differences in survival between predators.

To test the nonlethal effects of predator identity on tadpole behavioral responses (i.e., refuge use and spatial positioning), we used GLMM with a binomial family error model. In this analysis, predator identity was treated as a fixed effect, while temporal block and observation period were treated as random effects (to account for block effects and for the repeated observation periods). We compared all pairs of predators for each behavioral response variable using false discovery rate corrected pairwise comparisons (Stoffer, 2014).

## Results

### Survivorship

**Figure 1:**
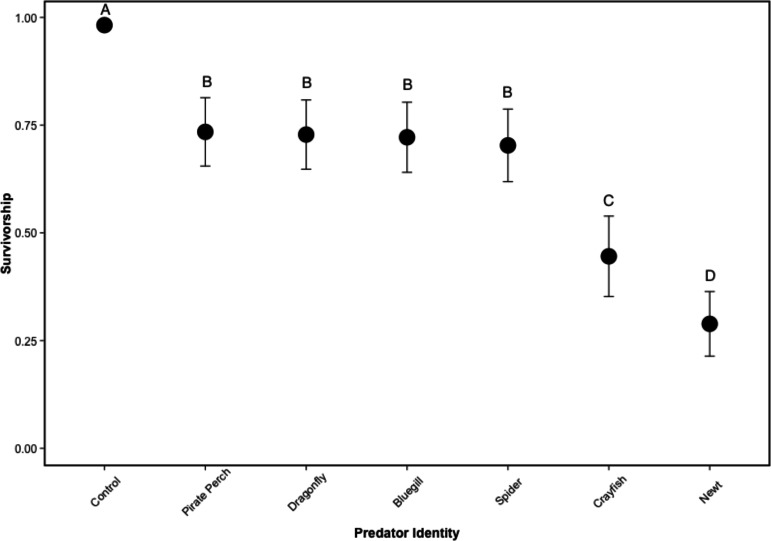
Tadpole survivorship with different predators. The model-generated mean survivorship of *Lithobates sphenocephalus* tadpoles in lethal predator treatments with 95% confidence intervals (${\chi }_{5}^{2}=127.43$, *P* < 0.001). Treatments that share the same letter are not significantly different, as determined by false discovery rate corrected post hoc analysis.

There was no mortality of prey or predators in the non-lethal predator treatments and the caged predators consumed all five tadpoles in every treatment. Similarly, there was 100% tadpole survival in all no-predator control treatments. In lethal predator treatments, predator identity significantly affected tadpole survival (${\chi }_{5}^{2}=127.43$, *P* < 0.001; *N* = 48; Fig. 1). Each predator significantly lowered tadpole survivorship compared to the no-predator control (bluegill: *Z* = 5.89, *P* < 0.001; crayfish: *Z* = 6.92, *P* < 0.001; dragonfly: *Z* = 5.87, *P* < 0.001; newt: *Z* = 7.49, *P* < 0.001; pirate perch: *Z* = 5.84, *P* < 0.001; spider: *Z* = 5.98, *P* < 0.001). The newt was the most lethal predator (0.40 ± 0.29 proportion surviving tadpoles) followed by the crayfish (0.45 ± 0.24). Newts were significantly more lethal than crayfish (*Z* = 2.98, *P* = 0.039), bluegill (*Z* = 7.69, *P* < 0.001), pirate perch (*Z* = − 7.89, *P* < 0.001), dragonfly (*Z* = 7.78, *P* < 0.001), and spider (*Z* = − 7.37, *P* < 0.001). Likewise, the crayfish ate significantly more tadpoles than the spider (*Z* = − 4.74, *P* < 0.001), pirate perch (*Z* = − 5.31, *P* < 0.001), bluegill (*Z* = 5.09, *P* < 0.001), and dragonfly predators (*Z* = − 5.02, *P* < 0.001). The amount of prey consumed by the bluegill, pirate perch, dragonfly, and spider did not differ from one another (*P* > 0.98 in all cases; Fig. 1).

### Tadpole refuge use

**Figure 2:**
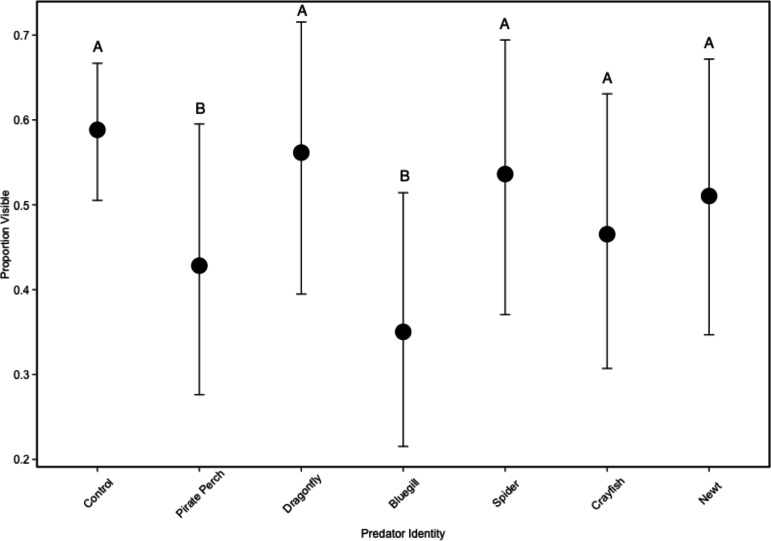
Tadpole visibility patterns with different predators. The model-generated mean visibility of *Lithobates sphenocephalus* tadpoles in the non-lethal predator treatments with 95% confidence intervals (${\chi }_{5}^{2}=127.43$, *P* < 0.001). Treatments that share the same letter are not significantly different from the control, as determined by false discovery rate corrected post hoc analysis.

In the non-lethal experiment, we detected that predator identity significantly affected refuge use (predator: ${\chi }_{6}^{2}\lt 0.001$; Fig. 2). There were fewer tadpoles visible in the bluegill and pirate perch predator treatments when compared to the no predator control (bluegill: *Z* = − 5.757, *P* < 0.001; pirate perch: *Z* = − 3.820, *P* = 0.003), while each of the other predators (newt, crayfish, spider, and dragonfly) did not differ from controls (Fig. 2). The fewest tadpoles were visible in the bluegill treatments with significantly fewer visible compared to the spider (*Z* = − 4.50, *P* < 0.001), newt (*Z* = − 3.89, *P* = 0.002), and to dragonfly predators (*Z* = − 5.11, *P* < 0.001). The pirate perch also reduced tadpole visibility and there were significantly fewer visible when compared dragonfly predators (*Z* = 3.18, *P* = 0.03).

### Tadpole spatial positioning

**Figure 3:**
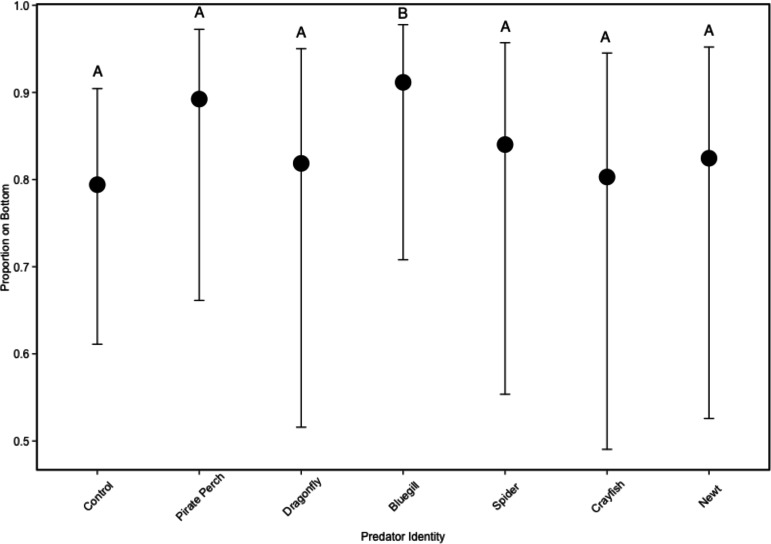
Tadpole spatial positioning patterns with different predators. The model-generated mean spatial position of *Lithobates sphenocephalus* tadpoles in the non-lethal predator treatments with 95% confidence intervals (${\chi }_{6}^{2}=18.44$, *P* < 0.005). Treatments that share the same letter are not significantly different from the control, as determined by false discovery rate corrected post hoc analysis.

In the non-lethal experiment, predator presence significantly affected the positioning of tadpoles in the water column (${\chi }_{6}^{2}=18.44$, *P* = 0.005; Fig. 3). The vertical distribution of tadpoles differed from the no-predator control in the bluegill treatment (*Z* = 3.57, *P* = 0.006). Tadpoles spent the most amount of time at the bottom of the tank in the bluegill treatment compared to the dragonfly (*Z* = 2.99, *P* = 0.04) and crayfish (*Z* = 3.37, *P* = 0.013) treatments.

## Discussion

Predator-naïve *L. sphenocephalus* tadpoles appear to have an innate response to fish predators but did not change spatial position or seek refuge in response to non-fish predators, including the two predators that consumed the most tadpoles, the newt and the crayfish. The presence of an innate response to the fish, but lack of detectable innate behavioral response to the other predators, may leave the tadpoles at greater risk of predation in early life stages. In time, the tadpoles may develop learned predator responses to the remaining predator species, but our results suggest that predator naïve tadpoles may incur high mortality and survival costs against newt and crayfish predators that could impact prey population sizes as well as community organization.

If prey have an innate response to some predators, but not others, one would expect that the innate response would have evolved to defend against either the most commonly encountered, or the most lethal, predators. In our experiment, this was not the case. Non-fish predators dominate most temporary ponds in our study area, including our collection sites. Furthermore, two of our non-fish predators (i.e., newts or crayfish) consumed a larger proportion of tadpoles in the lethal experiment than either of our fish predators (i.e., bluegill or pirate perch). Thus, either the presence of an innate response to fish effectively decreased the prey’s risk of being consumed by fish (resulting in lower levels of mortality with fish than with non-fish with whom prey lack an innate response) or predator-naïve *L. sphenocephalus* tadpoles are not always able to match the magnitude of behavioral response to the lethality of the predator.

Tadpoles may have developed innate responses to fish predators, even though they are not the most common or lethal predators, due to the length of co-evolutionary history together (DeSantis, Davis & Gabor, 2013). Tadpoles that live in more permanent bodies of water likely encounter fish predators regularly and these fish predators can decimate anuran egg or tadpole populations. Thus, an innate response to fish may have been highly adaptive if amphibian lineages throughout evolutionary history occurred with fish predators (Teplitsky, Plénet & Joly, 2003; Teplitsky, Plenet & Joly, 2004). Indeed, tadpoles of other species have been shown to produce innate adaptive responses to fish predators (Teplitsky, Plénet & Joly, 2003; Teplitsky, Plenet & Joly, 2004).

Tadpoles responded strongly and similarly to both of the fish species, bluegill and pirate perch. Recent work has suggested that pirate perch are able to mask cues to avoid detection by ovipositing amphibians (Resetarits & Binckley, 2013), but our results suggest that the same type of masking may not be effective in camouflaging their presence to coexisting larval prey since tadpoles both detected and responded strongly to pirate perch presence. Even if the kairomones produced by the pirate perch were masked, the digestive compounds produced by eating tadpoles made the pirate perch detectable to tadpoles. Further testing would be required to determine if pirate perch are able to avoid detection by other prey or predators of the pirate perch, but tadpoles can detect foraging pirate perch.

We considered the tadpoles in our experiment to be predator-naïve because they were collected as freshly laid eggs and hatched in water free from chemical cues. Some studies have shown that using eggs from lab-reared individuals is necessary to test innate predator response because even embryonic exposure to predator cue immediately after oviposition in ponds can cause later life stages to display generalized responses to some predators (Brown et al., 2013; Ferrari et al., 2009; Ferrari & Chivers, 2009). However, since we collected egg masses from fishless ponds, we would expect a higher response to the invertebrate predators rather than the fish if embryonic exposure had affected the eggs used in this study. Similarly, some studies have suggested that genetic maternal and paternal effects can impact anti-predator response (Stratmann & Taborsky, 2014). The tadpoles used in our experiment represented multiple clutches and clutches were divided evenly among aquaria. Thus, unless the mothers of all clutches were exposed to fish but not invertebrate predators (unlikely in our system), maternal effects would not explain our results.

The behavior of tadpoles in our study was highly variable, which may have limited our ability to detect differences in tadpole responses. For example, while tadpoles reduced visibility significantly more with the fish predators we noted that tadpoles also reduced visibility with the crayfish, albeit non-significantly. If tadpoles exhibited small changes in position or small increases in refuge use that we were unable to statistically detect, these behavioral responses would be so small as to be less likely to be biologically important. At the least, our results are clear that the strength of responses to fish are much greater than any changes in position or refuge use in response to fish. Moreover, we cannot exclude the possibility that our prey could have been responding to non-fish predators in some way that we did not measure (i.e., some way other than change in position or refuge use). Existing literature, however, describes the most common anti-predator response of tadpoles to be those responses that we measured (Skelly, 1992; Lefcort & Blaustein, 1995; Laurila, Kujasalo & Ranta, 1997; Parris, Reese & Storfer, 2006; Ferrari & Chivers, 2009).

Our results could be alternatively explained by differences in how the detectability of the six predator species was influenced by the experimental venue. For example, different predator species could differ in their ability to disseminate chemical cues or otherwise be detectable while in a cage. If chemical cues spread more widely from caged fish predators than caged non-fish predators, it could explain why fish predators induced stronger anti-predator responses in the prey. However, we do not think this explanation is likely as chemical cues have been shown to induce antipredator behaviors from over three meters away from the source and for up to 96 h following exposure (Turner & Montgomery, 2003; Peacor, 2006). Moreover, chemical cues from caged predators have been used in countless experiments to induce antipredator responses in tadpoles from a variety of predators (including invertebrate, fish, and amphibian predators) (Semlitsch & Reyer, 1992; Skelly, 1992; Lefcort & Blaustein, 1995; Laurila, Kujasalo & Ranta, 1997; Brown et al., 2006; Parris, Reese & Storfer, 2006; Ferrari & Chivers, 2009). As a result, we expect that cue from all predator species diffused to all sections of these tanks and did not degrade significantly over the 20-h trials. Finally, and perhaps most convincingly, behavioral observations taken during the lethal (uncaged predator) treatments showed comparable results to those shown in the caged predator treatments (Albecker, 2011). Thus, caging the predators does not seem to alter the behavioral impacts.

While it is possible that the experimental venue itself (i.e., aquarium) influenced either detectability or feeding rates of predators, there is no evidence that these impacts would be more substantial on some of these predator species over others. Every predator species consumed tadpoles during the experiment in the lethal treatments and in the cages, in every replicate of the experiment. In addition, there is a long history of experiments studying predator–prey interactions in the highly controlled setting of mesocosms (Chalcraft, Binckley & Resetarits, 2005) and producing comparable results to those from non-mesocosm settings (Skelly, 1992). The behavioral observations made here would not have been possible in a field setting.

## Conclusions

*L. sphenocephalus* tadpoles displayed an innate predator behavioral response to fish, but not non-fish predators. The presence of an innate response to the fish, but lack of detectable innate response to the other predators, may leave the tadpoles at greater risk from the most lethal predators until learning occurs. As a result, newly hatched tadpoles may incur high mortality in ponds with newt and crayfish predators that could impact prey population sizes, demography, and community organization.

## Supplemental Information

10.7717/peerj.1472/supp-1 Supplemental Information 1 Raw data_Survivor

10.7717/peerj.1472/supp-2 Supplemental Information 2 Raw data_Non lethal

## Additional Information and Declarations

### Field Study Permissions

The following information was supplied relating to field study approvals (i.e., approving body and any reference numbers): Animals were collected under North Carolina Wildlife Resources Commission Scientific Collection Permit (#10-SC00232).

### Data Availability

The following information was supplied regarding data availability: The raw data is supplied in the Supplemental Information. The data analysis code can be provided upon request, but it currently still needs to be cleaned up and properly annotated.
